# Association of systemic inflammatory factors with clinical outcomes in patients with autoimmune encephalitis at different clinical stages

**DOI:** 10.3389/fimmu.2025.1632690

**Published:** 2025-09-04

**Authors:** Bo Jin, Yiqun Duan, Wenxin Wu, Jing Hu, Xiaoyan Wu, Sheng Zhang, Huadong Wu, Thandar Aung

**Affiliations:** ^1^ Department of Neurology, Sir Run Run Shaw Hospital, Zhejiang University School of Medicine, Hangzhou, China; ^2^ Center for Rehabilitation Medicine, Department of Neurology, Zhejiang Provincial People's Hospital, Affiliated People's Hospital, Hangzhou Medical College, Hangzhou, Zhejiang, China; ^3^ Department of Neurology, Epilepsy Center, University of Pittsburgh Medical Center, Pittsburgh, PA, United States

**Keywords:** autoimmune encephalitis, systemic inflammatory factors, the clinical assessment scale for autoimmune encephalitis, disease severity, prognosis

## Abstract

**Objective:**

Our study aimed to explore the association of systemic inflammatory factors in relations to disease severity of the cell surface antibody-mediated autoimmune encephalitis (AE) across various stages.

**Methods:**

We retrospectively analyzed patients with AE from two hospitals between October 2016 and December 2023. Systemic inflammatory factors were measured at admission and discharge. Disease severity and prognosis were assessed using the clinical assessment scale for autoimmune encephalitis (CASE), and multivariate logistic regression analysis was used to identify associated risk factors.

**Results:**

A total 83 patients were enrolled. The CASE score and the modified Rankin Scale score were positively correlated at admission, discharge and follow-up (r=0.937, P < 0.001; r=0.910, P < 0.001; r=0.972, P < 0.001). Multivariate logistic regression analysis revealed that a higher systemic immune-inflammation index (SII) at admission (OR=27.617, 95% CI: 1.060–719.699, P=0.046) and an elevated platelet-to-lymphocyte ratio (PLR) at discharge (OR=11.373, 95% CI: 1.166–110.893, P=0.036) were independent risk factors for severe disease at admission and discharge, respectively. Additionally, a high neutrophil-to-platelet ratio (NPR) at either admission (OR=10.384, 95% CI: 2.036–52.958, P=0.005) or discharge (OR=5.714, 95% CI: 1.189–27.455, P=0.036) was associated with poor prognosis.

**Conclusions:**

SII and PLR were associated with disease severity, while NPR was a consistent predictor of poor long-term outcomes. These findings highlight the value of systemic inflammatory factors in monitoring disease progression and guiding treatment decisions in patients with AE mediated by cell surface antibody.

## 1 Introduction

Autoimmune encephalitis (AE) is a spectrum of autoimmune-mediated neurological diseases, characterized by an acute or subacute onset of memory dysfunction, psychiatric symptoms, involuntary movements, autonomic instability, impaired consciousness, and seizures (1, 2). Among various types of AE, antibody-mediated definite AE are the most well-distinguished and increasing incidences were reported (3, 4). Of all reported antibodies, cell surface AE- antibodies such as N-methyl-D-aspartate receptor (NMDAR), leucine-rich glioma-inactivated protein 1 (LGI1) and gamma-aminobutyric-acid B receptor (GABABR) are the most frequent antibodies (1, 3, 5). When compared to intracellular antibodies such as anti-Hu, cell-surface antibody-mediated definite AE responds well to immunology, especially early administration of immunotherapy (2). Still, even within the cell-surface antibody-mediated definite AE, many patients experience chronic symptoms, most notably cognitive impairment, that significantly affect quality of life (6–8). Cognitive dysfunction is not only a hallmark of AE but also one of the most disabling outcomes, often persisting despite apparent clinical improvement in other domains (8, 9). Therefore, accurate assessment of disease severity and reliable prognostic markers are essential to inform individualized treatment strategies and long-term care planning and currently evidence lacks established potential prognosticators.

Several potential blood biomarkers, such as neurofilament light chains, oligoclonal bands and antibody titers, have been studied during the acute phase of AE in an attempt to reflect disease severity and prognosis (9–13). However, it is not routinely implemented in clinical settings, primarily due to high testing costs and technical complexity. Recent evidence suggests an interplay between.

innate and adaptive immune systems in the pathophysiology of AE (14, 15). Thus, easily accessible systemic inflammatory factors, such as the neutrophil-to-lymphocyte ratio (NLR), monocyte-to-lymphocyte ratio (MLR), and systemic immune-inflammation index (SII), have been investigated to correlate with the severity and prognosis of AE (16–19). However, the prognostic value of these ratios remains inconsistent across studies due to heterogeneous study populations and variable study designs.

Although modified Rankin Scale (mRS) was commonly used to evaluate disease severity and prognosis in AE, it is relevant to point out that AE can present with a variety of clinical manifestations. As many manifestations of autoimmune encephalitis are non-motor in nature, the mRS may underestimate the true functional burden, in affected individuals. Recent study illustrates clinical assessment scale in autoimmune encephalitis (CASE) score has a higher sensitivity in detecting clinical changes compared to the mRS throughout the disease course (20–23).

Therefore, our study aimed to assess the value of various systemic inflammatory factors in predicting the severity of the definite AE patients related to cell surface antibodies using the CASE score.

## 2 Materials and methods

### 2.1 Patient selection and antibody testing

This retrospective study was approved by the institutional review boards of the Zhejiang Provincial People’s Hospital and the Sir Run Run Shaw Hospital of Zhejiang University. Written informed consent was obtained from all patients. We reviewed the consecutive medical charts of patients from the Zhejiang Provincial People’s Hospital and the Sir Run Run Shaw Hospital of Zhejiang University between October 2016 and December 2023. Diagnosis of AE was established using the diagnostic criteria established in 2016 (2). Serum and CSF samples were tested by cell-based assays for neuronal surface antibodies: NMDAR, LGI1, GABABR, GABA A receptor (GABAAR), contactin-associated protein-like 2 (CASPR2), a-amino-3-hydroxy-5-methyl-4-isoxazolepropinic acid receptor (AMPAR), dipeptidyl peptidase-like protein 6 (DPPX), IgLON5, metabotropic glutamate receptor 5 (mGluR5) and glycine receptor (GlyR). The exclusion criteria were as follows (1): patients with confounding conditions that could potentially affect white blood cell counts, including active infections, hematological disorders, and other systemic autoimmune diseases, were excluded (2). patients with alternative causes of encephalitis/encephalopathy, such as infectious, metabolic, endocrine, psychiatric, or rheumatologic diseases, were also excluded.

Age, sex, age at epilepsy onset, seizure frequency, cognitive symptoms, autoimmune comorbidities, autonomic dysfunction, electroencephalographic (EEG) findings, cerebral magnetic resonance imaging (MRI) findings, cerebrospinal fluid findings, and type of immunotherapy (steroid, intravenous immunoglobulin, rituximab, etc.) were collected.

### 2.2 Systemic inflammatory factors measurements

Venous blood samples were routinely collected for full blood count analysis within 24 hours after hospital admission and before discharge. NLR was defined as the ratio of the neutrophil count to the lymphocyte count. MLR was defined as the ratio of the monocyte count to the lymphocytes count. Neutrophil-to-platelet ratio (NPR) was defined as the ratio of the neutrophil count to the platelet count. Platelet-to-lymphocyte ratio (PLR) was defined as the ratio of the platelet count to the lymphocyte count. SII was defined as the product of platelet and neurophil counts divided by lymphocyte count [(platelet×neutrophil)/lymphocyte].

### 2.3 Evaluation of disease severity and prognosis of patients with AE

The participants were regularly followed by neurologists through telephone or in-person interviews conducted every three months. Relapses of AE were defined as recurrence or clear worsening of encephalitis symptoms occurring at least three months after complete remission or stable plateau of prior symptoms, accompanied by deterioration in ancillary testing findings. In the case of relapse or death, the time at which such an event occurs was considered their final follow-up. Based on previous studies (18, 24, 25), the CASE and mRS scores were independently accessed by two neurologists (B.J and S.Z) who were blinded to the diagnosis at the time of admission, discharge and each follow-up. All patients were divided into mild group (CASE ≤ 4) and severe group (CASE ≥ 5) according to the CASE score at admission and discharge. Poor prognosis was described as a CASE score of 5 or higher at the last follow-up visit. Patients who passed away during follow up were assigned a CASE score of 27, indicating maximum severity. Discrepancies in CASE scoring were re-reviewed by two neurologists to determine the final score. If the agreement could not be reached, the case was discussed in a group setting to achieve consensus.

### 2.4 Statistical analysis

Data were expressed as mean ± standard deviation or median and interquartile ranges (IQR). Categorical variables were analyzed using Chi-square test. If continuous variables were normally distributed, an independent sample t-test or one-way analysis of variance was used. If not, Mann–Whitney U tests or Kruskal–Wallis test was applied. Bonferroni correction was used for multiple comparisons when analyzing clinical characteristics among the three AE subgroups. Statistical significance was set to p < 0.017 (0.05/3). Spearman’s correlation analysis was performed to evaluate the correlation between the CASE score and mRS, the systemic inflammatory factors and disease severity (CASE score). Receiver operating characteristic (ROC) curve analysis was performed to assess the predictive performance for disease severity (CASE score) based on the systemic inflammatory factors. Cut-off values were estimated using the ROC curve, and the corresponding sensitivities and specificities were calculated based on the area under the curve (AUC). Systemic inflammatory factors levels were classified into two groups according to the cut-off values. To identify predictive factors for disease severity and long-term outcomes, variables with p values <0.05 in univariate analysis were included in multivariable logistic regression analyses. P<0.05 indicated statistical significance.

## 3 Results

### 3.1 Patient characteristics

During the study period, a total of 83 patients were enrolled. The median age was 52 ± 19.3 years old, and 52 (62.7%) were male. Among them, 35 (42.2%) patients were diagnosed with anti-NMDAR encephalitis, 24 (28.9%) with anti-LGl1 encephalitis, 13 (15.7%) with anti-GABABR encephalitis, eight with anti-CASPR2 encephalitis, two with anti-IgLON5, anti-DPPX and anti-mGluR5 encephalitis, and one with anti-AMPAR1 encephalitis. In a comparison of the most common subtypes of AE (anti-NMDAR, anti-LGI1, and others), anti-NMDAR encephalitis was found to be more prevalent in younger patients than in other subtypes (P < 0.001). Language problem was more common in anti-NMDAR encephalitis and other AE (P=0.001).

Three patients (one patient with anti-NMDAR encephalitis and two patients with anti-LGI1 encephalitis) were lost to follow-up, and 80 patients remained in the study group. Among 80 patients, 12 patients passed away, six patients experienced a relapse (median 13.5 months, IQR 9.3-32.8 months), and the remaining patients experienced a complete remission or plateauing from prior symptoms (median 36.5 months, IQR 20.0-50.5 months). The baseline clinical characteristics of the patients with different cell surface antibodies mediated AE were summarized in **Table 1**.

**Table 1:** Comparison of clinical characteristics among different AE subgroups.

	AE (n=83)	Anti-NMDAR encephalitis (n=35)	Anti-LGI1 encephalitis (n=24)	Other AE (n=24)	P
Male	52 (62.7%)	17 (48.6%)	14 (58.3%)	21 (87.5%)	0.009*^b^
Age at onset, years (IQR)	52.0 (32.0-63.0)	32.0 (22.0-52.0)	60.0 (45.0-66.8)	60.5 (48.5-68.5)	<0.001*^ab^
Duration from onset to Immunotherapy (IQR)	2.0 (1.0-5.0)	2.0 (1.0-5.0)	5.5 (2.0-21.0)	2.0 (2.0-4.0)	0.005*^a^
ICU admission	27 (32.5%)	14 (40.0%)	2 (8.3%)	11 (45.8%)	0.010*^ac^
Prodromal symptoms	21 (25.3%)	14 (40.0%)	2 (8.3%)	5 (20.8%)	0.019*^a^
Seizure	62 (74.7%)	25 (71.4%)	16 (66.7%)	21 (87.5%)	0.212
Psychiatric symptoms	62 (74.7%)	28 (80.0%)	18 (75.0%)	16 (66.7%)	0.511
Cognitive dysfunction	67 (80.7%)	29 (82.9%)	20 (83.3%)	18 (75.0%)	0.772
Language problem	47 (56.6%)	26 (74.3%)	6 (25.0%)	15 (62.5%)	0.001*^ac^
Dyskinesia/dystonia	19 (22.9%)	11 (31.4%)	7 (29.2%)	1 (4.2%)	0.034*^b^
Gait instability and ataxia	45 (54.2%)	22 (62.9%)	6 (25.0%)	17 (70.8%)	0.003*^ac^
Brainstem dysfunction	18 (21.7%)	12 (34.3%)	2 (8.3%)	4 (16.7%)	0.046^*^
Tumor	17 (20.5%)	5 (14.3%)	4 (16.7%)	8 (33.3%)	0.243
NLR at admission, median (IQR)	3.97 (2.79-7.75)	5.04 (2.44-9.05)	3.54 (2.80-5.20)	3.93 (2.93-8.11)	0.388
MLR at admission, median (IQR)	0.33 (0.23-0.49)	0.35 (0.20-0.55)	0.31 (0.22-0.42)	0.33 (0.25-0.50)	0.819
NPR at admission, median (IQR)	0.023 (0.018-0.035)	0.028 (0.019-0.037)	0.021 (0.015-0.027)	0.023 (0.019-0.039)	0.042*^a^
PLR at admission,median (IQR)	162.82 (125.71-207.48)	178.46 (110.32-261.68)	170.26 (139.56-206.15)	151.81 (127.30-195.38)	0.624
SII at admission,median (IQR)	926.98 (565.71-1497.60)	1187.26 (536.43-2239.63)	1028.11 (675.55-1339.58)	768.34 (524.23-1406.82)	0.383
CASE at admission, median (IQR)	6.0 (3.0-12.0)	10.0 (4.0-14.0)	4.5 (2.0-5.8)	7.0 (3.0-11.8)	0.018*^a^
CASE at discharge, median (IQR)	3.0 (2.0-6.0)	5.0 (2.0-8.0)	2.0 (1.0-3.0)	4.0 (2.3-6.0)	0.009*^a^
CASE at follow-up, median (IQR)	1.0 (0.0-3.0)	0.0 (0.0-3.0)	1.0 (0.0-2.0)	2.5 (0.0-27.0)	0.160

AE, autoimmune encephalitis; NMDAR, N-methyl-D-aspartate receptor; LGI1, leucine-rich glioma-inactivated 1; NLR, neutrophil-to-lymphocyte ratio; MLR, monocyte-to-lymphocyte ratio; NPR, neutrophil-to-platelet ratio; PLR, platelet-to-lymphocyte ratio; SII, systemic immune-inflammation index. CASE, clinical assessment scale for autoimmune encephalitis; IQR, interquartile ranges; P, comparison among the three groups of NMDA, LGI1, and other AE; a, NMDA vs. LGI1; b, NMDAR vs. other AEs; c, LGI1 vs. other AE; *indicates p value < 0.05; a, b, and c indicate Bonferroni correction p < < 0.017.

### 3.2 Validation of the CASE scale

**Figure 1:**
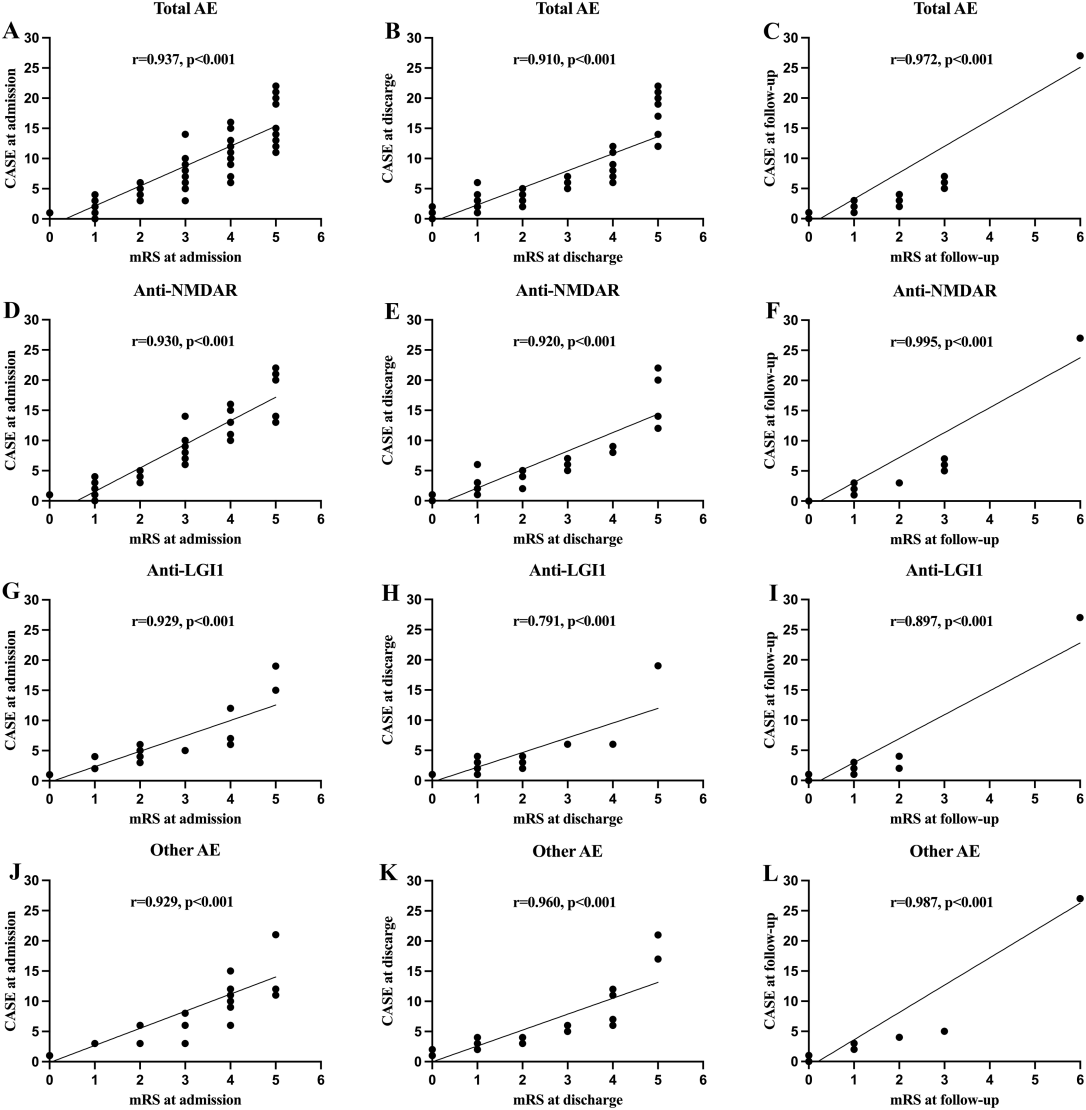
The correlations of clinical assessment scale for autoimmune encephalitis (CASE) score with the modified Rankin scale (mRS) at different stage. **(A–C)** Correlation between the CASE score in total patients with autoimmune encephalitis (AE); **(D–F)** Correlation between the CASE score in patients with anti-N-methyl-D-aspartate receptor (NMDAR) encephalitis; **(G–I)** Correlation between the CASE score in patients with anti-leucine-rich glioma inactivated 1 (LGI1) encephalitis; **(J–L)** Correlation between the CASE score in patients with other AE.

There was a strong correlation between CASE and mRS scores at admission, discharge and follow-up (**Figure 1**). The CASE score showed a broader range and more pronounced changes than the mRS within the same patient cohort, suggesting greater sensitivity in capturing disease severity and progression. We further stratified the correlation between the mRS and CASE scores among the three most common subtypes of AE (anti-NMDAR, anti-LGI1, and other AE) at admission, discharge, and follow-up, demonstrating strong correlations within each subgroup (**Figure 1**). Moreover, the CASE score also demonstrated more pronounced changes when compared to mRS scores across the three subtypes.

### 3.3 Systemic inflammatory factors and disease severity of AE at the time of admission

**Figure 2:**
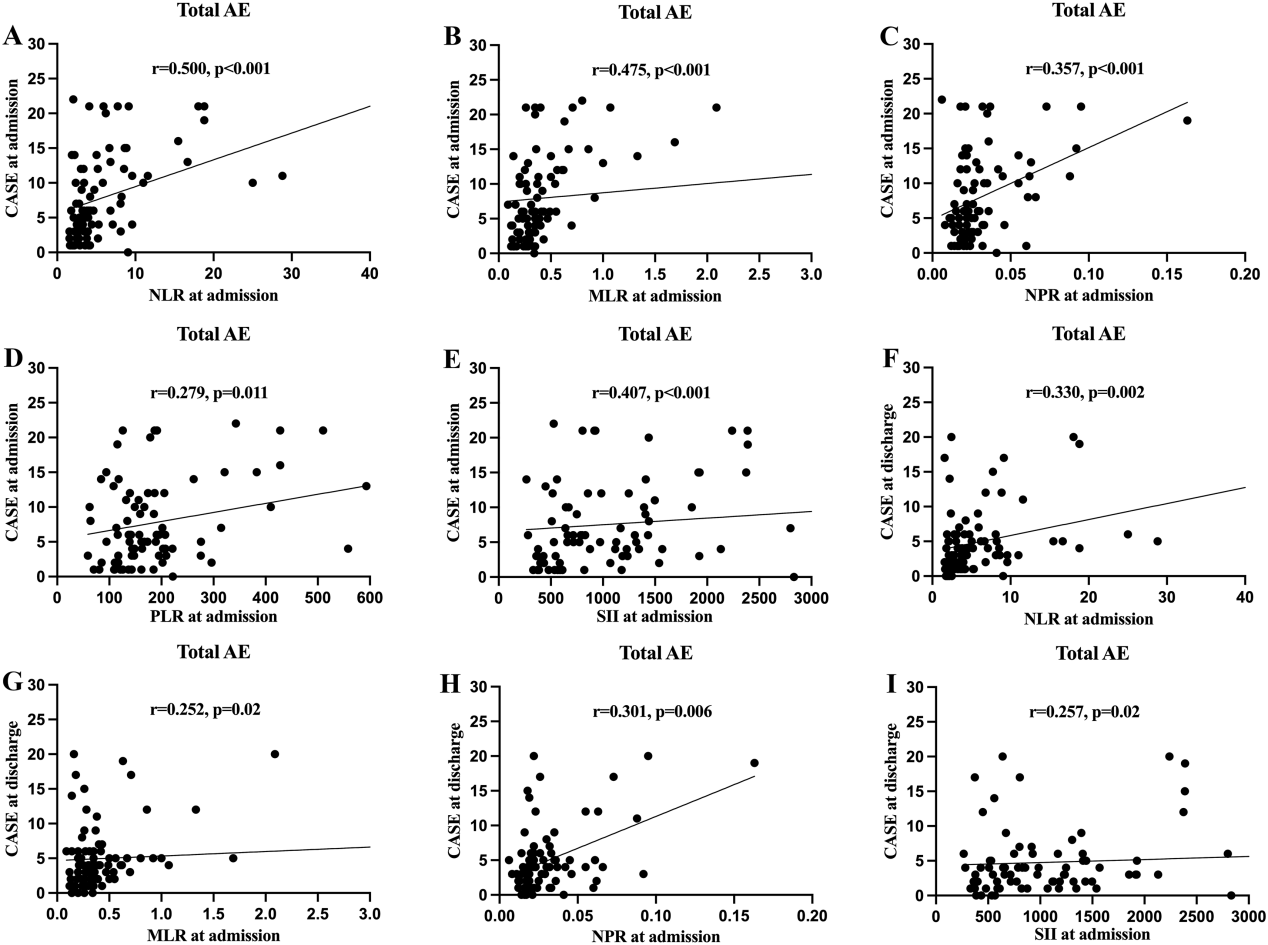
The correlations of clinical assessment scale for autoimmune encephalitis (CASE) score in total patients with autoimmune encephalitis (AE) with the systemic inflammatory factors at different stage. **(A)** Correlation between the CASE score at admission and neutrophil-to-lymphocyte ratio (NLR) at admission; **(B)** Correlation between the CASE score at admission and monocyte-to-lymphocyte ratio (MLR) at admission; **(C)** Correlation between the CASE score at admission and neutrophil-to-platelet ratio (NPR) at admission; **(D)** Correlation between the CASE score at admission and platelet-to-lymphocyte ratio (PLR) at admission; **(E)** Correlation between the CASE score at admission and systemic immune-inflammation index (SII) at admission; **(F)** Correlation between the CASE score at discharge and NLR at admission; **(G)** Correlation between the CASE score at discharge and MLR at admission; **(H)** Correlation between the CASE score at discharge and NPR at admission; **(I)** Correlation between the CASE score at discharge and SII at admission.

Based on the admission CASE scores, 30 patients (36.1%) were classified into the mild group, while 53 patients (63.9%) were placed in the severe group. Spearman’s correlation analysis showed that NLR, MLR, NPR, PLR and SII were positively correlated with admission CASE scores (**Figure 2**). ROC analysis was performed to assess the predictive value of NLR, MLR, NPR, PLR, and SII for disease severity. The AUC values for NLR, MLR, NPR, PLR, and SII were 0.666, 0.707, 0.631, 0.614, and 0.691, respectively. Details of the optimal cut-off values are provided in **Table 2**.

**Table 2:** ROC curve of the systemic inflammatory factors for the disease severity and prognosis.

Cut-off value		AUC (95%CI)	Sensitivity	Specificity	P value
The systemic inflammatory factors at admission and the disease severity of AE at admission					
NLR	4.19	0.666 (0.546-0.786)	56.6%	76.7%	0.012^*^
MLR	0.35	0.707 (0.594-0.820)	54.7%	86.7%	0.002^*^
NPR	0.025	0.631 (0.507-0.754)	52.8%	73.3%	0.049^*^
PLR	148.33	0.614 (0.486-0.741)	66.0%	56.7%	0.087
SII	610.08	0.691 (0.566-0.816)	84.9%	53.3%	0.004^*^
The systemic inflammation markers at admission and the disease severity of AE at discharge					
NLR	4.63	0.646 (0.521-0.771)	58.1%	71.2%	0.027^*^
MLR	0.62	0.632 (0.503-0.762)	32.3%	94.2%	0.044^*^
NPR	0.018	0.599 (0.476-0.722)	87.1%	32.7%	0.133
PLR	314.55	0.616 (0.486-0.747)	29.0%	94.2%	0.078
SII	1923.56	0.639 (0.510-0.768)	35.5%	92.3%	0.035^*^
The systemic inflammation markers at admission and the disease severity of AE at follow-up					
NLR	7.10	0.632 (0.474-0.790)	47.1%	79.4%	0.096
MLR	0.25	0.616 (0.476-0.755)	88.2%	34.9%	0.145
NPR	0.046	0.711 (0.552-0.871)	47.1%	95.2%	0.008^*^
PLR	134.62	0.346 (0.191-0.501)	52.9%	77.8%	0.053
SII	2239.63	0.592 (0.429-0.754)	29.4%	88.9%	0.249
The systemic inflammation markers at discharge and the disease severity of AE at discharge					
NLR	3.30	0.612 (0.485-0.740)	83.3%	39.1%	0.100
MLR	0.23	0.564 (0.433-0.694)	86.7%	26.1%	0.350
NPR	0.050	0.414 (0.286-0.543)	86.7%	30.4%	0.210
PLR	176.87	0.625 (0.495-0.756)	53.3%	71.7%	0.066
SII	1011.05	0.599 (0.469-0.730)	63.3%	56.5%	0.145
The systemic inflammation markers at discharge and the disease severity of AE at follow-up					
NLR	4.87	0.689 (0.552-0.825)	82.4%	55.4%	0.019^*^
MLR	0.35	0.706 (0.572-0.841)	82.4%	58.9%	0.010^*^
NPR	0.032	0.657 (0.509-0.804)	70.6%	60.7%	0.052
PLR	122.83	0.606 (0.457-0.754)	76.5%	44.6%	0.190
SII	1566.69	0.687 (0.534-0.840)	58.8%	78.6%	0.020^*^

(CASE score) of AE at different stage.

ROC, receiver operating characteristic; AE, autoimmune encephalitis; CASE, clinical assessment scale for autoimmune encephalitis; AUC, area under the curve; CI, confidence interval; NLR, neutrophil-to-lymphocyte ratio; MLR, monocyte-to-lymphocyte ratio; NPR, neutrophil-to-platelet ratio; PLR, platelet-to-lymphocyte ratio; SII, systemic immune-inflammation index. *indicates p value < 0.05.

Using univariate analysis, higher levels of NLR, MLR, NPR, PLR, and SII were significantly associated with more severe clinical status at admission (**Table 3**). To identify independent clinical predictors of disease severity, variables found to be significant in univariate analysis were included in the multivariate logistic regression model. The analysis revealed that psychiatric symptoms, language problem, gait instability and ataxia, and higher level of SII were independent risk factors for severe disease in patients with AE (**Table 3**). Patients were divided into two groups based on the optimal cut-off value of SII identified through ROC analysis. The corresponding clinical characteristics of the high and low biomarker groups are presented in **Supplementary Table 1**.

**Table 3:** Univariate and multivariable logistic regression analysis for the admission factors associated with the outcome (CASE score) at different stage.

	Univariate analysis			Multivariable logistic regression analysis	Univariate analysis			Multivariable logistic regression analysis	Univariate analysis			Multivariable logistic regression analysis
Characteristics at the time of admission	CASE ≤ 4 at admission (n=30)	CASE≥5 at admission (n=53)	P value	CASE≥5 at admission OR (95%CI), *P*	CASE ≤ 4 at discharge (n=52)	CASE≥5 at discharge (n=31)	P value	CASE≥5 at discharge OR (95%CI), *P*	CASE ≤ 4 at follow-up (n=63)	CASE≥5 at follow-up (n=17)	P value	CASE≥5 at follow-up OR (95%CI), *P*
Male	20 (66.7%)	32 (60.4%)	0.569		34 (65.4%)	18 (58.1%)	0.505		37 (58.7%)	14 (84.2%)	0.072	
Age at onset, years	51.0 (38.8-65.0)	52.0 (28.5-63.0)	0.694		51.0 (34.3-61.8)	52.0 (24.0-69.0)	0.992		48.0 (31.0-60.0)	65.0 (52.5-72.5)	0.002^*^	1.050 (1.009-1.092) 0.015^*^
Duration from onset to immunotherapy, days	2.5 (2.0-5.0)	2.0 (1.0-5.3)	0.694		3.0 (1.8-6.0)	2.0 (1.0-4.8)	0.104		2.0 (1.0-5.0)	3.0 (1.0-5.0)	0.866	
ICU admission	1 (3.3%)	26 (49.1%)	0.000^*^	477.120 (0.696-3.27*10^5) 0.064	7 (13.5%)	20 (64.5%)	0.000^*^	7.198 (2.016-25.702) 0.002^*^	20 (31.7%)	7 (41.2%)	0.466	
Prodromal symptoms	5 (16.7%)	16 (30.2%)	0.173		10 (19.2%)	11 (35.5%)	0.099		17 (27.0%)	4 (23.5%)	1.000	
Seizure	21 (70.0%)	41 (77.4%)	0.459		39 (75.0%)	23 (74.2%)	0.935		48 (76.2%)	13 (76.5%)	1.000	
Psychiatric symptoms	12 (40.0%)	50 (94.3%)	0.000^*^	142.679 (3.555-5726.190) 0.008^*^	33 (63.5%)	29 (93.5%)	0.002^*^	1.956 (0.257-14.873) 0.517	45 (71.4%)	14 (82.4%)	0.550	
Cognitive dysfunction	15 (50.0%)	52 (98.1%)	0.000^*^	24.739 (0.197-3103.360) 0.193	37 (71.2%)	30 (96.8%)	0.004^*^	1.482 (0.061-35.848) 0.809	49 (77.8%)	15 (88.2%)	0.539	
Language problem	3 (10.0%)	44 (83.0%)	0.000^*^	23.892 (1.940-294.243) 0.013^*^	20 (38.5%)	27 (87.1%)	0.000^*^	3.975 (1.002-15.762) 0.050	31 (49.2%)	14 (82.4%)	0.014^*^	3.752 (0.811-17.365) 0.091
Dyskinesia/ dystonia	7 (23.3%)	12 (22.6%)	0.943		11 (21.2%)	8 (25.8%)	0.626		15 (23.8%)	3 (17.6%)	0.832	
Gait instability and ataxia	4 (13.3%)	41 (77.4%)	0.000^*^	71.306 (3.129-1624.995) 0.007^*^	18 (34.6%)	27 (87.1%)	0.000^*^	2.574 (0.578-11.469) 0.215	31 (49.2%)	13 (76.5%)	0.045^*^	0.611 (0.085-4.407) 0.625
Brainstem dysfunction	0 (0.0%)	18 (34.0%)	0.000^*^	6.746*10^6 (0.000-NA) 0.998	4 (7.7%)	14 (45.2%)	0.000^*^	3.365 (0.777-14.574) 0.105	13 (20.6%)	5 (29.4%)	0.659	
Tumor	6 (20.0%)	11 (20.8%)	0.935		9 (17.3%)	8 (25.8%)	0.353		10 (15.9%)	7 (41.2%)	0.054	
NLR (>cut-off value)	7 (23.3%) (>4.19)	30 (56.6%) (>4.19)	0.003^*^	0.103 (0.008-1.278) 0.077	15 (28.8%) (>4.63)	18 (58.1%) (>4.63)	0.009^*^	0.499 (0.103-2.414) 0.387	13 (20.6%) (>7.10)	8 (47.1%) (>7.10)	0.059	4.909 (0.885-27.223) 0.069
MLR (>cut-off value)	4 (13.3%) (>0.35)	29 (54.7%) (>0.35)	0.000^*^	5.257 (0.335-82.431) 0.237	3 (5.8%) (>0.62)	10 (32.3%) (>0.62)	0.004^*^	7.096 (1.287-39.132) 0.024^*^	41 (65.1%) (>0.25)	15 (88.2%) (>0.25)	0.064	
NPR (>cut-off value)	8 (26.7%) (>0.025)	28 (52.8%) (>0.025)	0.021^*^	2.662 (0.072-98.627) 0.595	35 (67.3%) (>0.018)	27 (87.1%) (>0.018)	0.045^*^	2.266 (0.351-14.633) 0.390	3 (4.8%) (>0.046)	8 (47.1%) (>0.046)	0.000^*^	10.384 (2.036-52.958) 0.005^*^
PLR (>cut-off value)	13 (43.3%) (>148.33)	35 (66.0%) (>148.33)	0.044^*^	5.863 (0.289-118.842) 0.249	3 (5.8%) (>314.55)	9 (29.0%) (>314.55)	0.010^*^	1.586 (0.216-11.640) 0.650	49 (77.8%) (>134.62)	8 (47.1%) (>134.62)	0.029^*^	0.270 (0.051-1.442) 0.126
SII (>cut-off value)	14 (46.7%) (>610.08)	45 (84.9%) (>610.08)	0.000^*^	27.617 (1.060-719.699) 0.046^*^	12 (23.1%) (>1346.53)	15 (48.4%) (>1346.53)	0.017^*^	0.527 (0.034-8.108) 0.646	7 (11.1%) (>2239.63)	5 (29.4%) (>2239.63)	0.136	

CASE, clinical assessment scale for autoimmune encephalitis; NLR, neutrophil-to-lymphocyte ratio; MLR, monocyte-to-lymphocyte ratio; NPR, neutrophil-to-platelet ratio; PLR, platelet-to-lymphocyte ratio; SII, systemic immune-inflammation index. *indicates p value < 0.05.

### 3.4 Systemic inflammatory factors and disease severity of AE at the time of discharge

According to the CASE scores at the time of discharge, the patients were divided into a mild (52 patients, 62.7%) and severe disease (31 patients, 37.3%) groups. No statistically significant correlation was noted between systemic inflammatory factors and CASE score at discharge, using spearman’s correlation analysis. The optimal cut-off values were 3.3, 0.23, 0.05, 176.87 and 1011.05 for NLR, MLR, NPR, PLR, and SII respectively (**Table 2**).

Univariate analysis revealed higher levels of NLR and PLR were significantly associated with severe disease at the time of discharge (**Table 4**). Multivariate logistic regression analysis showed that psychiatric symptoms, cognitive dysfunction, language problem, gait instability and ataxia, and higher level of PLR as independent risk factors for disease severity in AE (**Table 4**).

**Table 4:** Univariate and multivariable logistic regression analysis for the discharged factors associated with the outcome (CASE score) at different stage.

	Univariate analysis			Multivariable logistic regression analysis	Univariate analysis			Multivariable logistic regression analysis
Characteristics at the time of discharge	CASE ≤ 4 at discharge (n=52)	CASE≥5 at discharge (n=31)	*P*	CASE≥5 at discharge OR (95%CI), *P*	CASE ≤ 4 at follow-up (n=63)	CASE≥5 at follow-up (n=17)	*P*	CASE≥5 at follow-up OR (95%CI), *P*
Male	34 (65.4%)	18 (58.1%)	0.505		37 (58.7%)	14 (84.2%)	0.072	
Age at onset, years	51.0 (34.3-61.8)	52.0 (24.0-69.0)	0.992		48.0 (31.0-60.0)	65.0 (52.5-72.5)	0.002^*^	1.051 (1.010-1.093) 0.013^*^
Duration from onset to immunotherapy, days	3.0 (1.8-6.0)	2.0 (1.0-4.8)	0.104		2.0 (1.0-5.0)	3.0 (1.0-5.0)	0.866	
ICU admission	7 (13.5%)	20 (64.5%)	0.000^*^	3.382 (0.539-21.219) 0.194	19 (29.2%)	7 (50.0%)	0.235	
Prodromal symptoms	10 (19.2%)	11 (35.5%)	0.099		17 (26.2%)	4 (28.6%)	1.000	
Seizure	38 (73.1%)	23 (74.2%)	0.911		47 (74.6%)	13 (76.5%)	1.000	
Psychiatric symptoms	16 (30.8%)	25 (80.6%)	0.000^*^	20.053 (2.550-157.687) 0.004^*^	28 (44.4%)	11 (64.7%)	0.138	
Cognitive dysfunction	25 (48.1%)	28 (90.3%)	0.000^*^	15.205 (1.152-200.678) 0.039^*^	41 (65.1%)	10 (58.8%)	0.634	
Language problem	11 (21.2%)	23 (74.2%)	0.000^*^	11.059 (1.260-97.024) 0.030^*^	21 (33.3%)	11 (64.7%)	0.019^*^	1.657 (0.349-7.857) 0.525
Dyskinesia/ dystonia	1 (1.9%)	4 (12.9%)	0.062		1 (1.6%)	3 (17.6%)	0.029^*^	97.856 (2.314-4140.184) 0.016^*^
Gait instability and ataxia	17 (32.7%)	28 (90.3%)	0.000^*^	22.946 (2.808-187.515) 0.003^*^	29 (46.0%)	15 (88.2%)	0.002^*^	20.956 (2.188-200.748) 0.008^*^
Brainstem dysfunction	0 (0.0%)	9 (29.0%)	0.000^*^	2.536*10^9 (0.000-NA) 0.998	5 (7.9%)	4 (23.5%)	0.170	
Tumor	9 (17.3%)	8 (25.8%)	0.353		10 (15.9%)	7 (41.2%)	0.054	
NLR (>cut-off value)	28 (60.9%) (>3.30)	25 (83.3%) (>3.30)	0.037^*^	0.802 (0.092-6.984) 0.842	25 (44.6%) (>4.87)	14 (82.4%) (>4.87)	0.006^*^	1.621 (0.145-18.111) 0.695
MLR (>cut-off value)	34 (73.9%) (>0.23)	26 (86.7%) (>0.23)	0.183		23 (41.1%) (>0.35)	14 (82.4%) (>0.35)	0.003^*^	2.815 (0.557-14.231) 0.211
NPR (>cut-off value)	14 (30.4%) (>0.050)	4 (13.3%) (>0.050)	0.087		22 (39.3%) (>0.032)	12 (70.6%) (>0.032)	0.023^*^	5.714 (1.189-27.455) 0.030^*^
PLR (>cut-off value)	13 (28.3%) (>176.87)	16 (53.3%) (>176.87)	0.028^*^	11.373 (1.166-110.893) 0.036^*^	31 (49.2%) (>122.83)	13 (76.5%) (>122.83)	0.119	
SII (>cut-off value)	20 (43.5%) (>1011.05)	19 (63.3%) (>1011.05)	0.091		12 (21.4%) (>1566.69)	10 (58.8%) (>1566.69)	0.003^*^	2.090 (0.254-17.228) 0.493

CASE, clinical assessment scale for autoimmune encephalitis; NLR, neutrophil-to-lymphocyte ratio; MLR, monocyte-to-lymphocyte ratio; NPR, neutrophil-to-platelet ratio; PLR, platelet-to-lymphocyte ratio; SII, systemic immune-inflammation index. *indicates p value < 0.05.

### 3.5 Systemic inflammatory factors at admission and disease severity of AE at discharge

We further investigated whether systemic inflammatory factors at admission could predict the disease severity at discharge. The NLR, MLR, NPR, and SII at admission were significantly and positively correlated with CASE scores at discharge (**Figure 2**). The optimal cut-off values were 4.63, 0.62, 0.018, 314.55 and 1923.56 for NLR, MLR, NPR, PLR, and SII respectively (**Table 2**). Univariate analysis showed higher levels of NLR, MLR, NPR, PLR, and SII at the time of admission were associated with severe disease severity at discharge (**Table 3**). Multivariate logistic regression analysis revealed that ICU admission and higher level of MLR were independent risk factors for disease severity of AE at discharge (**Table 3**). Patients were divided into two groups based on the optimal cut-off value of MLR. The clinical characteristics of the two groups are presented in **Supplementary Table 2**.

### 3.6 Systemic inflammatory factors at admission and disease severity of AE at follow-up

Based on the CASE score, 63 patients (78.8%) were in the mild disease group and 17 patients (21.2%) were in the severe group at the last follow-up. We further investigated whether systemic inflammatory factors at the time of admission could predict the disease severity at follow-up. Spearman’s correlation analysis showed that there was no significant correlation between systemic inflammatory factors at admission and CASE score at follow-up. The AUC values for NLR, MLR, NPR, PLR, and SII were 0.632, 0.616, 0.711, 0.346 and 0.592, respectively (**Table 2**).

Univariate analysis showed that compared with the good prognosis group, the poor prognosis group had higher elevated levels of NPR, PLR and SII at admission. Multivariate logistic regression analysis showed older age at onset, and higher level of NPR were independent risk factors for the poor prognosis of patients with AE (**Table 3**). Based on the optimal cut-off value of NPR, two groups of patients were stratified. The clinical features of both groups are outlined in **Supplementary Table 3**.

### 3.7 Systemic inflammatory factors at discharge and disease severity of AE at follow-up

We also investigated whether systemic inflammatory factors at the time of discharge could predict the disease severity at follow-up. No statistically significant correlations of systemic inflammatory factors at the time of discharge and CASE scores were noted. The optimal cut-off values for NLR, MLR, NPR, PLR, and SII were 4.87, 0.35, 0.032, 122.83, and 1566.69 respectively (**Table 2**). Univariate analysis showed that compared with the good prognosis group, the poor prognosis group had higher elevated level of NLR, MLR, NPR, and SII at discharge. Multivariate logistic regression analysis showed older age at onset, dyskinesia/dystonia, gait instability and ataxia, higher level of NPR were independent risk factors for the poor prognosis of AE patients (**Table 4**).

## 4 Discussion

Our study focused on the relationship between various systemic inflammatory factors and disease severity in patients with definite AE mediated by cell surface antibodies across different stages of the disease. Our findings indicate that there is strong correlation between systemic inflammatory factors and AE severity varies by disease stage. Our study also validates that the CASE scores are highly correlated with mRS scores and demonstrate strong validity for evaluating AE.

Increasing research has focused on systemic inflammatory factors for evaluating the inflammatory response and disease activity in several diseases, such as autoimmune disorders (26, 27), amyotrophic lateral sclerosis (28), myocardial infarction (29, 30), infective endocarditis (31). Recent studies illustrate innate and adaptive immune systems play more prominent and distinct role in AE (14, 15). Systemic inflammatory factors are robust stability and rarely influenced by physiological, pathological, or external factors, which affected by both innate immune response and adaptive immune response (14, 32). Several studies have examined the relationship between systemic inflammatory factors and disease severity or prognosis in AE, with inconsistent findings (16–19, 33). One study involving 121 patients with anti-NMDAR encephalitis showed that elevated NLR at admission was an independent risk factor for severe group (mRS>3) at admission (34). Another study involving 146 patients with AE showed that a high SII at admission predicted poor response to immunotherapy at 30 days assessed by mRS (19). In contrast, one retrospective study showed that higher levels of NLR and MLR at admission were associated with greater disease severity (CASE ≥ 5), but not with prognosis in 199 patients with AE (mRS > 2) (18). Similarly, another study showed that NLR, MLR, or PLR at admission were not associated with prognosis, as assessed by the mRS, in 34 patients with AE (35).

One major source of inconsistency across studies may be attributed to the use of different scoring systems for assessing AE severity. The CASE score has been proposed and validated to provide a more comprehensive evaluation of the diverse symptoms observed across different subtypes of AE and to offer a more accurate reflection of disease severity (20, 21, 23, 25). In our cohort of 83 patients, the most common clinical features were cognitive dysfunction, and seizures, consistent with prior studies showing cognitive dysfunction as hallmark of AE (8, 9). Among 67 patients with cognitive dysfunction, 63.7% of patients did not experience significant limitations in their daily activities. Of the 62 patients who experienced seizures, only seven patients had status epilepticus. These non-motor symptoms are often underrepresented by the mRS score. Additionally, similar to the previous data (20, 21, 23, 25), we observed a strong correlation between CASE and mRS scores at admission, discharge and follow-up. Thus, our study utilized the CASE score as the quantitative measure for assessing disease severity and prognosis. In contrast to previous studies that relied solely on admission data, our research incorporates both admission and discharge data to provide a more comprehensive assessment of disease progression and prognosis. Our findings are consistent with and extend prior studies that have examined the role of systemic inflammatory markers and clinical features in patients with AE mediated by cell surface antibodies. At admission, the higher level of SII level was an independent risk factor for severe disease. At discharge, the elevated PLR level was found to be an independent risk factor for severe disease. The elevated MLR level at admission predicted greater severity at discharge. Moreover, we are the first to demonstrate that a high NPR—whether at admission or discharge—was an independent risk factor for poor prognosis as assessed by the CASE score, a finding not previously reported in the literature. Neutrophils, as core components of innate immunity, can disrupt the function of blood-brain barrier (BBB) and increase its permeability by releasing a large number of pro-inflammatory factors such as interleukin 6, tumor necrosis factor alpha (TNF-a) and reactive oxygen species (ROS) (14, 36). Thus, neutrophils can contribute to significant inflammatory reactions. Platelets are involved in BBB integrity and cerebral microcirculation. Beyond these roles, platelets participate in inflammation and immune defense. Their granules contain a range of inflammatory mediators, cytokines, and growth factors, which are rapidly released upon activation (37, 38). High NPR values suggest persistent systemic inflammation and endothelial dysfunction (39), which may exacerbate the disease course and contribute to worse clinical outcomes in patients with AE. These findings indicate that systemic inflammatory factors may serve as potential indicators for assessing the disease progression of AE, providing important evidence for the treatment strategies.

Our study has several limitations. First, AE exhibits substantial subtype heterogeneity, with distinct pathogenic mechanisms, clinical phenotypes, and prognostic trajectories. Despite integrating data from two centers, the overall sample size, particularly within individual antibody-defined subgroups, remained limited, which may reduce the robustness of some statistical estimates. In particular, the wide confidence intervals observed in some multivariate models likely reflect the influence of small sample size. Second, despite the measurement of inflammatory factors within 24 hours of admission and discharge, inter-individual variability persisted due to the fluctuating nature of the disease state. Future studies with larger, multi-institutional cohorts are needed to validate these findings and allow more definitive stratified analyses by AE subtype.

## 5 Conclusion

In summary, our study shows that systemic inflammatory factors are associated with disease severity across all stages of AE mediated by cell surface antibodies. NPR, in particular, emerged as a novel and consistent predictor of poor long-term outcomes. These findings support the integration of systemic inflammatory factors with clinical assessment to monitor disease progression, and identify high-risk patients early to guide treatment decisions.

## Data Availability

The original contributions presented in the study are included in the article/**Supplementary Material**. Further inquiries can be directed to the corresponding author.
