# Supplementary material for: Association of systemic inflammatory factors with clinical outcomes in patients with autoimmune encephalitis at different clinical stages
Source: Front Immunol. 2025 Sep 4;16:1632690. doi: 10.3389/fimmu.2025.1632690 (PMC12443571; doi:10.3389/fimmu.2025.1632690)
Supplement: Supplementary file 1 [file Table1.docx]

| Supplementary table 1. Clinical characteristics of patients with AE in different SII at admission. | | | | |
| --- | --- | --- | --- | --- |
|  | AE (n=83) | Low SII  (n=23) | High SII  (n=60) | *P* |
| Male | 52 (62.7%) | 13 (56.5%) | 39 (65.0%) | 0.475 |
| Age at onset, years  (IQR) | 52.0 (32.0-63.0) | 54.0 (35.0-67.0) | 51.0 (31.3-63.0) | 0.413 |
| ICU admission | 27 (32.5%) | 5 (21.7%) | 22 (36.7%) | 0.194 |
| Prodromal symptoms | 21 (25.3%) | 4 (17.4%) | 17 (28.3%) | 0.305 |
| Seizure | 62 (74.7%) | 16 (69.6%) | 46 (76.7%) | 0.505 |
| Psychiatric symptoms | 62 (74.7%) | 10 (43.5%) | 52 (86.7%) | <0.001^*^ |
| Cognitive dysfunction | 67 (80.7%) | 15 (65.2%) | 52 (86.7%) | 0.058 |
| Language problem | 47 (56.6%) | 7 (30.4%) | 40 (66.7%) | 0.003^*^ |
| Dyskinesia/  dystonia | 19 (22.9%) | 6 (26.1%) | 13 (21.7%) | 0.668 |
| Gait instability and ataxia | 45 (54.2%) | 10 (43.5%) | 35 (58.3%) | 0.224 |
| Brainstem dysfunction | 18 (21.7%) | 3 (13.0%) | 15 (25.0%) | 0.373 |
| Tumor | 17 (20.5%) | 5 (21.7%) | 12 (18.4%) | 1.000 |

AE, autoimmune encephalitis; SII, systemic immune-inflammation index; IQR, interquartile ranges; *indicates p value < 0.05.
