# Supplementary material for: Association of systemic inflammatory factors with clinical outcomes in patients with autoimmune encephalitis at different clinical stages
Source: Front Immunol. 2025 Sep 4;16:1632690. doi: 10.3389/fimmu.2025.1632690 (PMC12443571; doi:10.3389/fimmu.2025.1632690)
Supplement: Supplementary file 2 [file Table2.docx]

| Supplementary table 2. Clinical characteristics of patients with AE in different MLR at admission. | | | | |
| --- | --- | --- | --- | --- |
|  | AE (n=83) | Low MLR  (n=68) | High MLR  (n=15) | *P* |
| Male | 52 (62.7%) | 44 (64.7%) | 8 (53.3%) | 0.410 |
| Age at onset, years  (IQR) | 52.0 (32.0-63.0) | 51.0 (32.3-61.8) | 63.0 (26.0-74.0) | 0.320 |
| ICU admission | 27 (32.5%) | 20 (29.4%) | 7 (46.7%) | 0.230 |
| Prodromal symptoms | 21 (25.3%) | 17 (25.0%) | 4 (26.7%) | 1.000 |
| Seizure | 62 (74.7%) | 48 (70.6%) | 14 (93.3%) | 1.000 |
| Psychiatric symptoms | 62 (74.7%) | 47 (69.1%) | 15 (100%) | 0.009^*^ |
| Cognitive dysfunction | 67 (80.7%) | 52 (76.5%) | 15 (100%) | 0.064 |
| Language problem | 47 (56.6%) | 34 (50.0%) | 13 (86.7%) | 0.009^*^ |
| Dyskinesia/  dystonia | 19 (22.9%) | 14 (20.6%) | 5 (33.3%) | 0.317 |
| Gait instability and ataxia | 45 (54.2%) | 32 (47.1%) | 13 (86.7%) | 0.005^*^ |
| Brainstem dysfunction | 18 (21.7%) | 13 (19.1%) | 5 (33.3%) | 0.298 |
| Tumor | 17 (20.5%) | 14 (20.6%) | 3 (20.0%) | 1.000 |

AE, autoimmune encephalitis; MLR, monocyte-to-lymphocyte ratio; IQR, interquartile ranges; *indicates p value < 0.05.
