# Supplementary material for: Association of systemic inflammatory factors with clinical outcomes in patients with autoimmune encephalitis at different clinical stages
Source: Front Immunol. 2025 Sep 4;16:1632690. doi: 10.3389/fimmu.2025.1632690 (PMC12443571; doi:10.3389/fimmu.2025.1632690)
Supplement: Supplementary file 3 [file Table3.docx]

| Supplementary table 3. Clinical characteristics of patients with AE in different NPR at admission. | | | | |
| --- | --- | --- | --- | --- |
|  | AE (n=83) | Low NPR  (n=70) | High NPR  (n=13) | *P* |
| Male | 52 (62.7%) | 43 (61.4%) | 9 (69.2%) | 0.758 |
| Age at onset, years  (IQR) | 52.0 (32.0-63.0) | 50.0 (31.8-63.0) | 58.0 (48.5-73.0) | 0.095 |
| ICU admission | 27 (32.5%) | 20 (28.6%) | 7 (53.8%) | 0.106 |
| Prodromal symptoms | 21 (25.3%) | 17 (24.3%) | 4 (30.8%) | 0.730 |
| Seizure | 62 (74.7%) | 53 (75.7%) | 9 (69.2%) | 0.730 |
| Psychiatric symptoms | 62 (74.7%) | 50 (71.4%) | 12 (92.3%) | 0.168 |
| Cognitive dysfunction | 67 (80.7%) | 54 (77.1%) | 13 (100%) | 0.064 |
| Language problem | 47 (56.6%) | 36 (51.4%) | 11 (84.6%) | 0.027^*^ |
| Dyskinesia/  dystonia | 19 (22.9%) | 16 (22.9%) | 3 (23.1%) | 1.000 |
| Gait instability and ataxia | 45 (54.2%) | 34 (48.6%) | 11 (84.8%) | 0.017 |
| Brainstem dysfunction | 18 (21.7%) | 14 (20.0%) | 4 (30.8%) | 0.465 |
| Tumor | 17 (20.5%) | 16 (22.9%) | 1 (7.7%) | 0.286 |

AE, autoimmune encephalitis; NPR, neutrophil-to-platelet ratio; IQR, interquartile ranges; *indicates p value < 0.05.
